# Making Better Use of Population Health Data for Community Health Needs Assessments

**DOI:** 10.5334/egems.305

**Published:** 2019-08-20

**Authors:** Michael A. Stoto, Mary V. Davis, Abby Atkins

**Affiliations:** 1 Georgetown University, US; 2 Health Resources in Action, US

**Keywords:** Community Health Needs Assessment (CHNA), Community health improvement process, Community health assessment, Shared measurement systems, Population health data

## Abstract

**Research Objective:**

Non-profit hospitals are required to work with community organizations to prepare a Community Health Needs Assessment (CHNA) and implementation strategy (IS). In concert with the health care delivery system’s transformation from volume to value and efforts to enhance multi-sector collaboration, such community health improvement (CHI) processes have the potential to bridge efforts of the health care delivery sector, public health agencies, and community organizations to improve population health. Having a shared measurement system is critical to achieving collective impact, yet despite the availability of community-level data from a variety of sources, many CHI processes lack clear, measurable objectives and evaluation plans. Through an in-depth analysis of ten exemplary CHI processes, we sought to identify best practices for population health measurement with a focus on measures for needs assessments and priority setting.

**Study Design:**

Based on a review of the scientific literature, professional publications and presentations, and nominations from a national advisory panel, we identified 10 exemplary CHI processes. Criteria of choice were whether (1) the CHIs articulate a clear definition of intended outcomes; (2) clear, focused, measurable objectives and expected outcomes, including health equity; (3) expected outcomes are realistic and addressed with specific action plans; and (4) whether the plans and their associated performance measures become fully integrated into agencies and become a way of being for the agencies. We then conducted an in-depth analysis of CHNA, IS, and related documents created by health departments and leading hospitals in each process.

**Population Studied:**

U.S. hospitals.

**Principal Findings:**

Census, American Community Survey, and similar data are available for smaller areas are used to describe the populations covered, and, to a lesser extent, to identify health issues where there are disparities and inequities.

Common data sources for population health profiles, including risk factors and population health outcomes, are vital statistics, survey data including BRFSS, infectious disease surveillance data, hospital & ED data, and registries. These data are typically available only at the county level, and only occasionally are broken down by race, ethnicity, age, poverty.

There is more variability in format and content of ISs than CHNAs; the most developed models include population-level goals/objectives and strategies with clear accountability and metrics. Other hospital IS’s are less developed.

**Conclusions:**

The county is the unit of choice because most population health profile data are not available for sub-county areas, but when a hospital serves a population more broadly or narrowly defined, appropriate data are not available to set priorities or monitor progress.

Measure definitions are taken from the original data sources, so comparisons across measures is difficult. Thus, although CHNAs cover many of the same topics, the measures used vary markedly. Using the same community health profile, e.g. County Health Rankings, would simplify benchmarking and trend analysis.

Implications for Policy or Practice: It is important to develop population health data that can be disaggregated to the appropriate geographical level and to groups defined by race and ethnicity, socioeconomic status, and other factors associated with health outcomes.

## Introduction

Among the many provisions of the *Affordable Care Act* (ACA) that aim to improve population health, one stands out as having the potential for bridging the efforts of the health care delivery sector, public health agencies, and other community organizations to improve population health outcomes. Under IRS §501(r) regulations, all non-profit hospitals are required to work with health departments and other community organizations to conduct a Community Health Need Assessment (CHNA) at least every three years and adopt an implementation strategy (IS) describing how identified needs will be addressed. These regulations have the potential to refocus some of the “community benefits” that hospitals are required to spend (estimated at $62 billion in 2011) towards improving population health [1,2].

As we describe in more detail in a companion paper [3], although the CHNA requirements offer an extraordinary *promise* for advancing population health through multi-sector collaboration to build health partnerships [4], evaluations of current efforts suggest that the new IRS regulations [5] do not yet seem to have not achieved their potential in most communities.

To follow this lead, we conducted an in-depth analysis of ten exemplary community health improvement (CHI) processes to identify best practices. This analysis is based on an in-depth analysis of 10 exemplary CHI processes identified based on a review of the scientific literature, professional publications and presentations, and nominations from a national advisory panel. We take as our starting point that population health is a shared responsibility, and that managing it requires two different measurement strategies. First, there should be a shared community health profile to identify common priorities for outcomes with standard measures that facilitate comparisons with similar communities, changes over time, and benchmarking with other communities and external standards. Second, implementation plans for hospitals and other entities should include pre-specified performance measures to align efforts and to ensure accountability for the organizations’ actions to address community health improvement [6,7,8]. A companion paper describes our methodology and addresses current practices in performance measurement [3]. This paper focuses on measures for community health needs assessments and priority setting.

## Definition of the population served

Every one of the 10 CHI processes we reviewed (see Table 1) had created a shared community health assessment (CHA) or CHNA that aligned the efforts of one or more hospitals, local health departments (LHD), and other stakeholders. In 6 of the 10 processes (Ashland-Boyd, Cecil, King, Monroe, San Francisco, Yellowstone), these reports were prepared by or with significant input from the LHD, demonstrating health departments strengths and potential contribution both as a convener and a source of data expertise. These CHAs/CHNAs typically serve as a data repository and are used for many purposes beyond priority setting per se, especially grant writing.

**Table 1:** CHNA coverage and community description.

Process	Responsible for CHNA	Hospitals	Geography	Community description

Ashland-Boyd County	Ashland-Boyd County HD	*Bon Secours Our Lady of Bellefonte Hospital*, serves patients in 6 KY, 2 OH, and 2 WV Counties; *King’s Daughter Medical Center* serves patients in the same counties as the CHA	Ashland-Boyd County CHA includes Boyd, Carter, Greenup County, KY and Lawrence County, OH	Census/ACA type data at the county level for Boyd County only
Baton Rouge	Baton Rough mayor’s office	*Baton Rouge General Medical Center* + 4 non-profit and 1 for-profit hospitals; Baton Rouge General CHNA lists EBR, Livingston and Ascension parishes as in the “primary market”	East Baton Rouge Parish (EBR, county equivalent), which includes city of Baton Rouge; Baton Rouge General CHNA	Census/ACA type data at the county level for East Baton Rouge Parish only; maps of Community Needs Index (CNI) by Zip code for all EBR, Livingston and Ascension parishes
Bexar County	The Health Collaborative	*Methodist Hospital* serves patients in 13 counties; other hospitals in THC include *Baptist Health System, CHRISTUS Santa Rosa Health System*, and *University Health System*	Bexar County, with data on 8 subcounty areas for many indicators	Census/ACA type data at the county level, life expectancy by Zip code, both for Bexar County only
Cecil County	Cecil County HD & Union Hospital	*Union Hospital of Cecil County*	Cecil County, MD	Census/ACA type data at county level
King County	King County Hospitals for a Healthier Community, facilitated by Seattle-King County HD	17 hospitals including *Swedish Seattle*, which serves both King and Snohomish counties	King County	Maps showing Health, Housing and Economic Opportunity Measure index and other Census/ACA data by Census tract for King County only
Monroe County	Monroe County HD, 4 hospitals, & Finger Lakes Health System Agency	*University of Rochester Medicine* & *Rochester Regional Health* (2 hospitals each)	Monroe County, with data by census tract for a few indicators	Census/ACA type data for Monroe County level & City of Rochester
Pittsburgh	UPMC system (Allegheny County CHNA not referenced)	*UPMC Children’s Hospital of Pittsburgh*	Allegheny County (although only 37% of UPMC Children’s Hospital of Pittsburgh patients live there	Census/ACA type data at the county level; map showing federally-designated as Medically Underserved Areas (MUAs)
Rutland County	Rutland Regional Medical Center	*Rutland Regional Medical Center*, which also serves portions of southern and north central Vermont and Washington County, NY	Rutland County	Population counts for Rutland County and Health Service Area; assorted data for “targeted populations” (seniors, individuals in poverty, adults with substance abuse issues, youth and adults who are overweight or obese)
San Francisco	San Francisco Health Improvement Partnership (SFHIP), San Francisco county HD lead in secondary data	*Kaiser Foundation Hospital-San Francisco, Saint Francisco Memorial Hospital (Dignity Health), St. Mary’s Medical Center (Dignity Health), California Pacific Medical Center (4 campuses, Sutter Health)*, & the *Chinese Hospital*	San Francisco County	Census/ACA type data at the county level; selected indicators available by neighborhood in SFHIP Strategic Priorities report
Yellowstone County	Professional Research Consultants, Inc. (PRC), for The Alliance, including Yellowstone County HD	*St. Vincent Healthcare*	Yellowstone County Zip codes (some spilling over to other counties)	Census/ACA type data at the county level

For example, Public Health Seattle King County (PHSKC) facilitates the shared CHNA process, including serving as the backbone organization for this work. Each entity may choose to use all or part of the shared CHNA data and supplement those data with additional data specific to the populations the hospital or health system serves. Seattle Children’s Hospital created its own community health assessment by tailoring the joint CHNA to focus on pediatric and adolescent populations and includes the communities in the area it serves beyond King County. Each hospital and health system prepared its own community benefits reports and PHSKC prepared its own Community Health Improvement Plan. According to those we interviewed,

- “It’s successful if the hospitals understand and use the data, especially the disparities we see in the data.”
- “The collaborative is really about the data. There are common indicators that we all need to know that define a healthy community.”

The population of interest in these reports is typically defined as *one* county (see Table 1). For small counties with one hospital (Cecil, Yellowstone), and for some large counties with multiple hospitals (Bexar, Monroe, San Francisco), this seems to represent a close relationship to the populations that the hospitals serve.

However, several CHNAs we reviewed covered more than one county and did not match well with the hospital service areas.

- The Ashland-Boyd County CHA covers four counties in two states (Boyd, Carter and Greenup in Kentucky and Lawrence in Ohio), but only presents data for Boyd County. One hospital in this region serves patients in the same four counties, while a second hospital serves patients in 10 counties in three states. Both hospital CHNAs, however, include data for Carter, Greenup, and Lawrence Counties that is not included in the Ashland-Boyd County CHA, along with data on Boyd County.
- The city of Baton Rouge CHNA covers only East Baton Rouge (EBR) parish (the equivalent of a county, which includes more territory than the city of Baton Rouge), whereas one of the hospital’s “primary market” includes EBR and two other counties.
- The Seattle-King County CHNA covers King County only, although one hospital serves both King (1.9 million population) plus neighboring Snohomish (0.7 million population) county.
- The Rutland CHNA is based on data primarily for Rutland County. Counties are not a major administrative unit in most of New England, and the Rutland Regional Medical Center also serves other portions of southern and north central Vermont and Washington County, New York.

Two of the hospitals in our sample focused health improvement efforts on more targeted populations. The CHNA of Children’s Hospital of Pittsburgh of UPMC (University of Pittsburgh Medical Center) describes the hospital as “a primary source of care for children and adolescents in western Pennsylvania.” The hospital’s CHNA, however, includes only data for Allegheny County, where 37 percent of hospital’s patients reside, and these data relate to individuals of all ages, not just children. The CHNA prepared by the Allegheny County Health Department is not referenced in Children’s Hospital CHNA. The San Francisco Health Improvement Partnership CHNA relates to San Francisco County. Saint Francis Memorial Hospital’s IS, on the other hand, focuses exclusively on the Tenderloin District, without offering population data to support this choice.

## Population descriptions and profiles used in CHNAs

The CHNAs and CHAs in our sample typically include descriptions of the population in demographic and socioeconomic terms: population size, broken down by race, ethnicity, poverty/income, and so on. The Monroe County CHNA includes separate data for City of Rochester, but the rest do not distinguish sub-county areas in their demographic descriptions. These demographic data may appear in a background section or as “social determinants of health” in a population health profile.

The demographic descriptions are typically based on Census, American Community Survey, and similar data sets. Only four of the 10 CHI processes we reviewed display data by Zip code or smaller geographic area. The Baton Rouge CHNA includes maps showing life expectancy by Zip code, and the Pittsburgh CHNA includes a map showing federally-designated Medically Underserved Areas. The King County CHNA includes maps showing the Health, Housing and Economic Opportunity Measure index and other Census/ACA data by Census tract, and the San Francisco Health Improvement Partnership (SFHIP) Strategic Priorities report includes Census-type data for selected indicators available by neighborhood. Data of this sort are potentially useful for identifying geographic areas of greater need or health issues where there are disparities and inequities, but the written reports do not clearly indicate that they are being used in this way.

As summarized in Table 2, the 10 CHI processes we reviewed drew on a wide variety of data sources to construct a population health profile. These included:

**Table 2:** Population health profile and community input data.

Process	Population health profile	Community input survey data

Ashland-Boyd County	BRFSS, vital statistics, & other public health data at the county level (some via County Health Rankings & Roadmaps)	NA
Baton Rouge	BRFSS, vital statistics, & other public health data for EBR at the county level (via County Health Rankings & Roadmaps with LA and US benchmarks)	Refers to YMCA’s Community Healthy Living Index, but no data shown
Bexar County	BRFSS, vital statistics, hospitalization & other public health data at the county level	Online survey of 310 individuals residing in most of the zip codes in Bexar County
Cecil County	BRFSS, vital statistics, & other public health data at the county level (many referencing MD State Health Improvement Process indicators and goals) for identified priorities only	Online survey of 506 individuals identified through multiple sources
King County	Maps showing disparities in life expectancy by neighborhood, table with leading causes of death by age, both for King County only; BRFSS, vital statistics, hospitalization & other public health data at the county level, variously broken down by race & ethnicity, socioeconomic status, income, 4 regions within King County	NA
Monroe County	Monroe County Adult Health Survey (similar to BRFSS), vital statistics, hospitalization (from Statewide Planning and Research Cooperative Systems (SPARCS) files), & other public health data at the county level (many referencing NY State Prevention Agenda Dashboard)	NA
Pittsburgh	Selected data for identified priorities only	NA
Rutland County	BRFSS, vital statistics, & other public health data at the county level (many via County Health Rankings & Roadmaps) for identified priorities only	Online survey of 673 individuals identified through multiple sources
San Francisco	Kaiser Foundation Hospital uses Kaiser Permanente data platform with 150 publicly available indicators covering social and economic factors; health behaviors; physical environment; clinical care; and health outcomes; other hospitals incorporate and build on SFHIP Strategic Priorities report	NA
Yellowstone County	BRFSS, vital statistics, & other public health data at the county level using standard Professional Research Consultants, Inc. (PRC) dashboard with trends and benchmarks	Survey conducted by PRC (random sample with n = 400, weighted to census demography, similar surveys in previous years)

- Vital statistics (cause-specific death rates, birth certificate data, etc.). Because vital statistics are based on a complete count of births and deaths, they are available for smaller geographic areas, but they were rarely used in this way.
- Survey data (health-related behaviors such as diet & physical activity; the self-reported prevalence of chronic conditions such as diabetes; health care access and utilization, etc.). These data are drawn from the CDC Behavioral Risk Factor Surveillance System (BRFSS), similar adult health surveys conducted by state or local health departments, state-based youth risk behavior surveillance surveys, and the National Immunization Survey, etc.
- Infectious disease surveillance data drawn from the state health department or the CDC.
- Hospital and emergency department (ED) data. The type of data available and used varies by state.
- Data from the federal agencies such as the Health Resources and Services Administration (HRSA) and the Centers for Medicare and Medicaid Services (CMS) on health resources, access to care, utilization, etc.
- Cancer, diabetes, and HIV/AIDS registries.
- Existing reports such as CDC and state health department reports on sexually transmitted infections, diabetes, and tobacco; *Kentucky Health Facts* and *Kentucky Youth Advocates* (Ashland-Boyd); and *Healthy Vermonters 2020* (Rutland).

These and other data drawn from websites or on-line data systems such as:

- the County Health Rankings (Ashland-Boyd, Baton Rouge, Rutland)
- the New York State Prevention Agenda Dashboard (Monroe), the Maryland State Health Improvement Process indicators (Cecil)
- Healthy People 2020 (Pittsburgh & Rutland)
- KIDS Count Data, the Dartmouth Atlas, a dashboard developed by Professional Research Consultants (PRC), a consulting firm that conducts CHNAs for many hospitals in the U.S. (Yellowstone)
- Kaiser Permanente’s data system-wide data platform (Kaiser Foundation Hospital, Saint Francisco).

The CHAs/CHNAs we reviewed varied markedly in their analysis of historical trends, use of comparisons to other communities, and other “benchmarks.” Communities’ ability to compare or benchmark against other communities is determined by the source of the data. Some data platforms such as the County Health Rankings & Roadmaps, the New York and Maryland data systems, and the PRC and Kaiser Permanente dashboards allow for trend analysis and comparisons with other jurisdictions usually within the same state. Otherwise, few temporal or geographic comparisons are included in CHNAs.

The availability of data on disparities in health factors and outcomes by race and ethnicity, socioeconomic factors, and other “upstream” factors also depends on data source. The King County CHNA, for instance, includes a map showing disparities in life expectancy by neighborhood. Otherwise, such disparities are rarely described.

The presentation of population health profile data varies markedly among the processes we reviewed. Some CHAs/CHNAs used a dashboard format, often with benchmarks and trends used to help identify priorities (King, Monroe, Yellowstone). Others used a narrative format with graphs used to summarize the situation for issues already chosen as priorities (Cecil, Pittsburgh, San Francisco, Rutland). For others, data are used in a more *ad hoc* way.

Information of this sort is typically labeled as “secondary data” to distinguish it from surveys or focus groups conducted specifically for the CHNA. However, only 4 of the 10 CHI processed we studied included data from a special purpose community survey on community concerns. Two conducted a survey with a representative sample of community members (Yellowstone, using PRC questionnaire, n = 400; Bexar, n = 310), and two others used a convenience sample with individuals identified through multiple sources (Cecil, n = 506; Rutland, n = 673).

## Discussion

The creation of a shared CHNA for a defined geographic area is a success in and of itself: it demonstrates a degree of collaboration between hospitals, health departments, and other entities that our environmental scan suggests is not common [9].

Some of those interviewed for the case studies regarded the CHNA as a shared resource, useful for hospitals, health departments, and community-based organizations’ proposal writing and other purposes. For others, sharing the cost of producing the CHNA was a primary motivator. In addition to producing a valuable product, working together on a shared CHNA represents an important aspect of a shared measurement system, and facilitates the development of a common agenda, collaboration and stakeholder engagement. Indeed, at one of the sites, hospital interviewees indicated that the opportunity to work with other hospitals on issues that affected their shared communities has been an important motivator for their organization’s continued engagement, and that a shared CHNA facilitated this collaboration.

Logically, it makes sense to identify priorities for a geographically-defined population such as the residents of a specific county for three reasons: 1) doing so facilitates collaboration among hospitals and other partners, 2) the most important problems may be in populations not currently well-served by specific or all hospitals, and 3) analyzing county population health data allows for the identification of socio-economically disadvantaged geographical areas within the county that might not be well-served by current hospital service patterns.

With few exceptions, however, the CHNAs we reviewed chose a single county as their unit of analysis even though the hospitals participating in the CHI process often serve smaller, or larger, populations than a county. Choosing a single county as the focus of a CHNA reflects, in part, LHDs’ involvement in their creation (even when hospitals served patients in multiple counties, typically only one LHD was involved in preparing the CHNA). It should be noted, however, that the IRS regulations require each individual hospital’s CHNA to describe the community the hospital serves and identify that community’s significant health needs. When a hospital serves a population more broadly or narrowly defined than a county, the CHNA based on county data does not contain appropriate data to set priorities or monitor progress. Moving forward, the hospitals in a city or county might agree on a common community definition that allows for population-wide interventions and priority setting. Individual hospitals could choose target areas for their own implementation strategies reflecting a targeted focus to address disparities [10].

Consistent with the county as the unit of choice, the data in the CHNAs in our sample are typically presented in no finer detail than the county level. This is in part a statistical limitation, since for some counties the sample size available in the BRFSS and other surveys is too small to allow precise estimates below the county level. Even rates based on complete counts (e.g. deaths for vital statistics, cases for infectious disease reports), there may be too few events to support sub-county estimates.

Although all of the CHNAs in our sample include some demographic and socio-economic data to describe the population, only two of the CHI processes (King and San Francisco counties) explicitly present socio-economic data by neighborhood. Furthermore, the choice of measures included seems to be *ad hoc*. Other sub-county data sources that could have been used to identify disadvantaged populations for specific attention are Dignity Health’s Community Needs Index [11], the University of Wisconsin Area Deprivation Index [12], and other data available through Community Commons [13] and the American Academy of Family Physicians’ HealthLandscape [14].

In the CHNAs we reviewed, we found that population health outcomes and risk factors were only broken down by race, ethnicity, age, poverty, and so on in some of these documents. Consequently, it is difficult to identify specific target populations based on health factors or outcomes. Here again, there are alternatives that were not employed. At least for the more urban areas, CDC‘s 500 Cities project [15] provides model-based estimates at the Census tract level for 500 largest U.S. cities. UDS Mapper, developed by the American Academy of Family Physicians [16], uses data within the Uniform Data System (UDS) from the federal Section 330 Health Center Program for small area estimates of health care utilization by underserved populations.

Many of the CHNAs we reviewed reported hospitalization and ED utilization data, but typically only at the county level. Nagasako and colleagues have shown that it is possible to extend the County Health Rankings & Roadmaps population health measurement model to the ZIP code level using widely available hospital and census-derived data sources [17]. Similarly, Gross and colleagues describe how the Camden Coalition of Healthcare Providers partnered with the three health systems providing emergency and inpatient care in Camden, New Jersey, to create an all-payer hospital claims data set that provides unique insights into the health status, health care utilization patterns, and hospital costs on the population level [18].

Even at the county level, communities’ ability to put their population health measures into context is limited. Although the CHNAs we studied cover many of the same health topics, the specific measures used vary markedly. Definitions are taken from original data sources, so comparisons across measures within a CHNA are difficult. Counties are typically compared to state or national levels only, as opposed to “peer” counties with similar characteristics. Trend data are only sometimes available. Consequently, hospitals’ ability to “benchmark” (i.e. compare their results to other similar hospitals) is limited. Although local buy in is important, it might be more useful for planning purposes if every community used the same health profile to simplify benchmarking and trend analysis. The model used by County Health Rankings & Roadmaps [19], which provides consistent data for all U.S. counties for a series of population health outcomes and factors, is an obvious starting point. This database includes a tool for identifying peer counties [20]. Nagasako and colleagues have described how the same concepts can be monitored with sub-county estimates based on hospitalization and census data [17].

**Figure 1:**
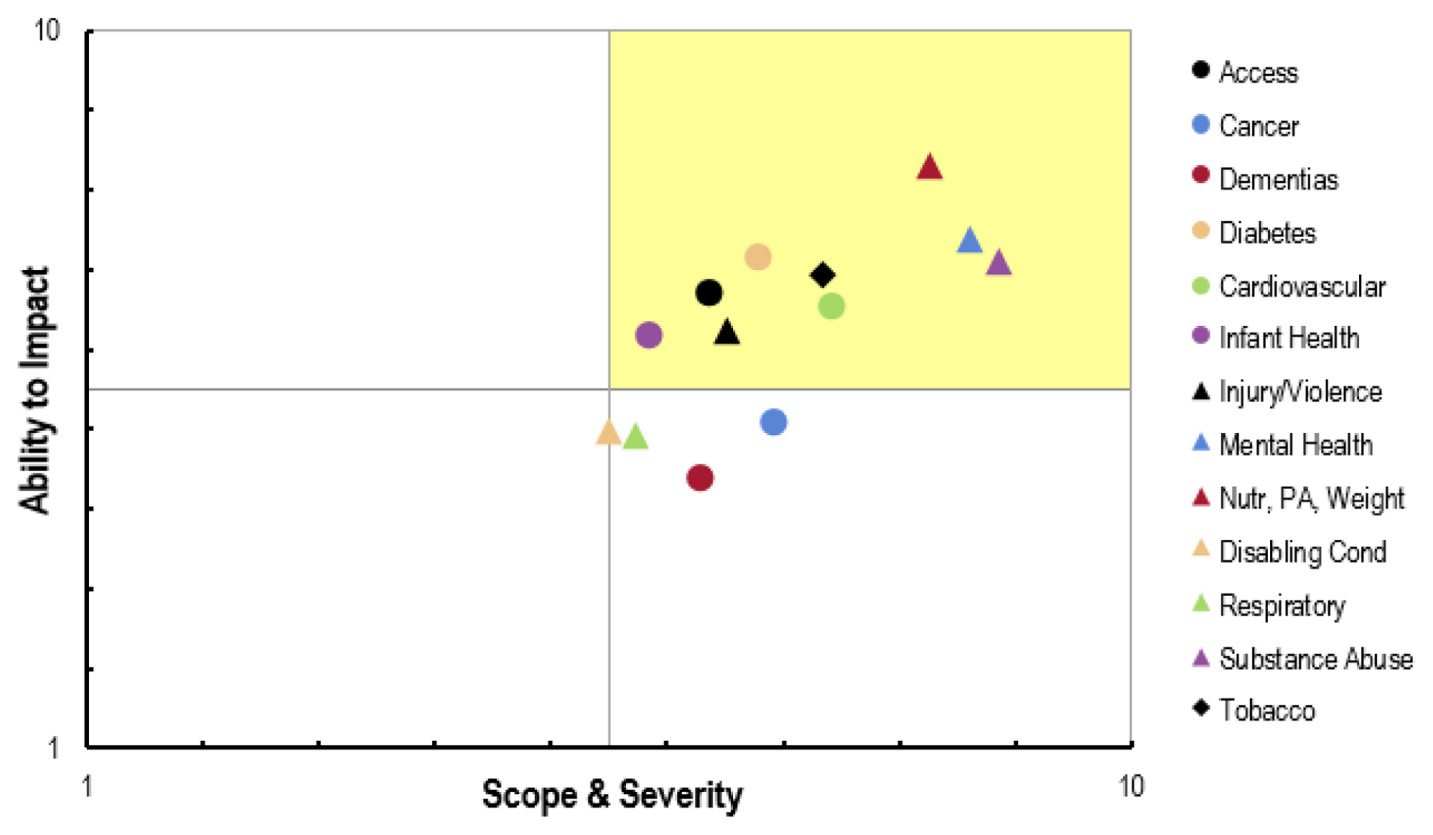
Potential priorities ranked according to scope & severity and ability to impact. *Source:* 2016–17 Community Health Needs Assessment Report, Yellowstone County, Montana [21].

For many hospitals, “doing a CHNA” means conducting some kind of survey, usually with a small, non-representative sample and an *ad hoc* questionnaire. Borrowing language from the IRS guidance, this is regarded as “primary” data, as opposed to the other types of data used in CHNAs, which are regarded as “secondary” data. Surveys of this type can be useful for obtaining perceptions and concerns, but cannot provide objective information about population health factors or outcomes. Even if they are targeted at the hospital’s users, it is usually not a representative sample, and the sample size is usually too small to obtain an acceptable level of precision. If the goal of these efforts is to get informed community input, it could be more useful to convene a group of individuals (opinion leaders or representative patients, representing different constituents) and ask them react to community input together with “objective” data. For example, as a starting point for their priority setting process, Monroe County reviewed data from the New York State Prevention Agenda County Dashboard and identified indicators that were worse than the NYS average and/or they did not meet the Prevention Agenda targets. Similarly, Yellowstone County convened group of community stakeholders (representing a cross-section of community-based agencies and organizations) to evaluate, discuss and prioritize health issues for community, based on findings of CHNA. Participants were asked to evaluate each health issue along two criteria: (1) scope & severity and (2) ability to impact. Project staff they used a scatter plot of these results (Figure 1) to choose priorities.

## Conclusions

A recent study conducted for the Collective Impact Forum by ORS Impact and Spark Policy Institute [22] examined 25 collective impact initiatives across the U.S. with the goal of surfacing insights about when and how collective impact achieves impact. One of the key findings was that the successful sites more frequently: (1) implemented data strategies, (2) included the shared measurement system in their explanation for how change happened, and (3) prioritized data-related early and/or systems changes as a critical part of their contribution story. In this context, a “shared measurement system” was defined as

- agreed-upon common indicator(s) established to consistently track progress across time
- functional approach and system to collect, store, analyze, and report valid and reliable data
- output/results of shared measurement system are actionable for data use (timely, meaningful, relevant, sensitive to change, targeted to goal, etc.)
- not necessarily a common database or warehouse.

The Collective Impact Forum report also found that shared measurement systems are not always present, but when they are it is tied to having a common agenda and mutually reinforcing activities [22] (two of the other five components of Collective Impact).

The Collective Impact Forum report also reported common challenges associated with shared measurement systems that are consistent with the problems we identified in our analysis. One set of challenges involved what data are—or are not—collected. One of the common challenges was the relative lack of data at the “right level”:

Site visit sites indicate data is sometimes not useful when it is not at the right level (e.g., community level versus case level). Several initiatives are struggling with gaps in the data, such as having data from some geographic areas and not others or some types of institutions and not others. In the context of CHI processes, the parallel issue is the need for more data at the sub-county level [22].

Many of the issues identified in the Collective Impact Forum report [22] were reflected in our case studies. For instance, some of the CHNAs in our sample do not systematically include sub-county data to justify their chosen target populations. Communities should consider the argument represented in the Massachusetts Attorney General’s Office requirement that hospitals choose a “target area” for the focus of their implementation strategy that is not necessarily the geographic hospital service area or patient care population. Rather, it could be a “disadvantaged population” defined by a combination of three factors:

- geographic boundary, e.g., a city, town, county or several contiguous municipalities, not necessarily limited by the hospital’s direct service area;
- demographic factors, e.g., a community may be defined by
  1. the low or moderate-income persons who are uninsured
  2. the elderly; or
  3. pregnant women of low or moderate income; and
- health status, e.g., focusing on the prevalence of a particular disease, such as HIV, sexually transmitted infections, diabetes, or cardio-vascular disease, within disadvantaged populations in the service area [23].

The ten CHI processes in our analysis were chosen as exemplars, but yet on the whole did not demonstrate the characteristics of mature shared measurement systems. One possible explanation is that, despite living in a “big data” era, we don’t have consistent population health data below the county level. This is especially true for behavioral risks, self-reported outcomes, and similar survey-based measures. Furthermore, although hospital data are theoretically available in smaller units, judging by their absence in the CHI processes we reviewed, they are not consistently available to the teams developing CHNAs and ISs.

This suggests that two things are needed to help ensure that the CHNA process helps to transform health care systems to improve outcomes. First, it is important to develop population health data that can be disaggregated to the appropriate geographical level and to groups defined by race and ethnicity, socioeconomic status, and other factors associated with health outcomes. As we describe in the Discussion, there are indeed many existing sources of survey, administrative, and other data that can be synthesized with information on the social context and health care resources at the geographical level that is necessary.

Second, it is important to develop practitioners’ knowledge and skills needed to use it population health data effectively. Here we can build on the growing effective data use in value-based purchasing arrangements such as Accountable Care Organizations and Accountable Health Communities. It also suggests a role for public health agencies in advancing data availability and use. Some LHDs already having the required data and expertise, and others must develop it. LHDs also can serve as a neutral party to convene hospitals and other agencies to share data.
